# Effect of 5'UTR introns on gene expression in *Arabidopsis thaliana*

**DOI:** 10.1186/1471-2164-7-120

**Published:** 2006-05-19

**Authors:** Betty YW Chung, Cas Simons, Andrew E Firth, Chris M Brown, Roger P Hellens

**Affiliations:** 1 Biochemistry Department, University of Otago, Dunedin, New Zealand; 2 HortResearch, Auckland, New Zealand; 3 Institute of Molecular Biosciences, Brisbane, Australia; 4 Bioscience Institute, University College Cork, Cork, Ireland

## Abstract

**Background:**

The majority of introns in gene transcripts are found within the coding sequences (CDSs). A small but significant fraction of introns are also found to reside within the untranslated regions (5'UTRs and 3'UTRs) of expressed sequences. Alignment of the whole genome and expressed sequence tags (ESTs) of the model plant *Arabidopsis thaliana *has identified introns residing in both coding and non-coding regions of the genome.

**Results:**

A bioinformatic analysis revealed some interesting observations: (1) the density of introns in 5'UTRs is similar to that in CDSs but much higher than that in 3'UTRs; (2) the 5'UTR introns are preferentially located close to the initiating ATG codon; (3) introns in the 5'UTRs are, on average, longer than introns in the CDSs and 3'UTRs; and (4) 5'UTR introns have a different nucleotide composition to that of CDS and 3'UTR introns. Furthermore, we show that the 5'UTR intron of the *A. thaliana *EF1α-A3 gene affects the gene expression and the size of the 5'UTR intron influences the level of gene expression.

**Conclusion:**

Introns within the 5'UTR show specific features that distinguish them from introns that reside within the coding sequence and the 3'UTR. In the EF1α-A3 gene, the presence of a long intron in the 5'UTR is sufficient to enhance gene expression in plants in a size dependent manner.

## Background

Introns, first discovered in 1977 [1], are genomic sequences that are removed from the corresponding RNA transcripts of genes. The most abundant class are spliceosomal introns, which are found in the nuclear genomes of all characterized eukaryotes, and rely on spliceosomes – a complex that comprises five RNAs and hundreds of proteins – for successful splicing from RNA transcripts [2,3]. There are two types of spliceosomal introns: (1) U2 introns, which are the most abundant and are spliced by the U2-type spliceosome, and (2) the rarer U12 introns (< 0.4%), which are spliced by the less abundant U12-type spliceosome [2]. In this paper we consider only plant U2 spliceosomal introns.

A growing number of plant expression studies on chimeric RNA have demonstrated that such intron sequences can enhance the level of protein expression, a phenomenon termed Intron-Mediated Enhancement (IME) [4-10]. Inclusion of an intron in the 5' region of a gene, either in the 5'UTR or fused to the 5' portion of the coding sequence, leads to enhanced RNA levels [11-15]. While the degree of expression enhancement varies for each intron, up to a 1000-fold increase in protein accumulation has been reported [16]. The alteration in RNA and protein accumulation is known to act post-transcriptionally [17]. Nonetheless, the intrinsic determinants of 5'UTR IME in plants, especially those within the intron itself, remain poorly defined.

The plant *Arabidopsis thaliana *has a compact genome and generally small introns [18], consistent with the proposed correlation between intron size and genome size [19,20]. On the other hand, the length of intron contributes to the energetic cost of transcription, which is proportional to the length of the transcript produced [21]. Therefore, the fact that a significant number of 5'UTRs contain introns suggests that these, like coding sequence introns, may be functionally important. Mechanistically it is possible that the 5'UTR introns are involved in IME and act in the nucleus [8], and it has been proposed that IME results from synergistic interactions between the factors involved in the various steps of gene expression from transcription to translation [22]. The elevated translational efficiency is most likely due to an increased in the affinity of mRNA to ribosomes via their interactions with exon junction complexes (EJCs), which are deposited on the mRNA 20–24 nucleotides upstream of introns during splicing [23-26].

Studies on plant introns have revealed a strong nucleotide bias toward T proximal to the AG intron acceptor site, and throughout the intron there is an A/T bias relative to the adjacent exon [27]. While these nucleotide biases are believed to be required for efficient intron recognition and splicing in coding region introns [28], for introns that reside within the non-coding regions, there is no nucleotide bias that distinguishes intron from exon sequence. To date there are no studies on the statistical properties of 5'UTR introns on the genomic scale in multicellular eukaryotes. Here we present a comprehensive bioinformatic analysis of nucleotide composition, intron-position, and intron-length distribution of all the annotated *A. thaliana *5'UTR U2 introns supported by EST and cDNA data. Our results show that, firstly, the density of introns in the 5'UTRs is similar to that in the CDSs but much higher than that in the 3'UTRs; secondly, introns within the 5'UTR are not randomly distributed along the UTR but are more likely to be located closer to the ATG; thirdly, the introns that reside within the 5'UTR are, on average, significantly larger than the average intron found in both the CDS and 3'UTR; and finally, the sequences around the splicing junctions show distinct nucleotide bias that distinguish them from CDS and 3'UTR introns. Our findings indicate that 5'UTR introns may be subject to different selective forces from the introns in CDSs and 3'UTRs, possibly due to a specific regulatory role in gene expression. These observations are exposed in the well-annotated and relatively compact *Arabidopsis *genome.

To complement the bioinformatic analysis, an experimental analysis of the *A. thaliana *gene *EF1α-*A*3 *– which has an intron-containing 5'UTR – was undertaken in order to investigate what influence 5'UTR introns have on gene expression, and how this is affected by intron length. We confirm that the presence of the 5'UTR intron in *EF1α-*A*3 *increases gene expression [13-15] levels 3-fold in transient assays and over 10-fold in stable transgenic plants. In addition, a deletion series based on the intron length showed that the expression level is dependent either on intron length or distributed motifs dispersed throughout the 5' region of the intron.

## Results and discussion

### Bioinformatics analysis

The presence, frequency, length distributions, and structure of introns and exons have been extensively studied [29-35]. While it is known that the presence of a 5'UTR intron can enhance gene expression [36], not much is known about the underlying mechanism for this phenomenon. In this study, an extensive bioinformatic analysis of *A. thaliana *introns was undertaken, using the TAIR (The *Arabidopsis *Information Resource) database [37]. This study focuses particularly on the length, position and nucleotide composition of CDS and UTR introns, in order to characterize the differences between 5'UTR, CDS and 3'UTR introns.

#### Analysis of 5' and 3' untranslated regions

There are 32,955 annotated protein-coding genes supported by EST sequences in the TAIR database. Of these, 18,285 include both the 5'UTR and the 3'UTR, 527 contain the 5'UTR but not the 3'UTR, 1,979 contain the 3'UTR but not the 5'UTR, and 12,171 contain neither. Considerably more genes were found to lack 5'UTR annotation than 3'UTR annotation, probably due to the directional construction of cDNA libraries from the polyA tail. A number of the annotated 5'UTR sequences are expected to be only partial sequences; however, this is not expected to greatly affect the conclusions of this paper (see below). Many protein-coding genes (72.0%) contain introns. While the non-coding regions are less likely to include introns, these are more commonly found in the 5'UTR: 19.9% of annotated 5'UTRs contain introns; by comparison only 5.6% of 3'UTRs are annotated to include introns. The high number of intron-containing 5'UTRs cannot be explained by the 5'UTR lengths, as the average 5'UTR that has been delimited by EST and/or cDNA sequences is considerably shorter than the average 3'UTR region (5'UTR: median = 99 nucleotides, LQ (lower quartile) = 56 nucleotides, UQ (upper quartile) = 175 nucleotides; 3'UTR: median = 208 nucleotides, LQ = 154 nucleotides, UQ = 283 nucleotides). Indeed, Table 1 shows that the average number of introns per nucleotide is, 1.6 × 10^-3^, 2.7 × 10^-3 ^and 2.9 × 10^-4 ^in 5'UTRs, CDSs and 3'UTRs, respectively. Since these intron densities are normalized by the total length of sequence, incomplete UTR annotation should not greatly bias these results. Thus the intron density in 5'UTRs is ~60% of the intron density in CDSs and ~5.5 times the intron density in 3'UTRs. In mammals, a marked under-representation of introns in the 3'UTRs may be explained by the requirements for nonsense-mediated decay (NMD) [38,39]. The corresponding under-representation measured here in *A. thaliana *may indicate that plants utilize a similar NMD pathway.

**Table 1:** Table showing statistics of 5' UTR, CDS and 3' UTR.

	Number of sequences	Sequences with introns	Total bases (genomic)	intron/sequence	Number of introns/nucleotide (mRNA)
5'UTR	18,812	3,738	2.6 × 10^6^	0.23	1.6 × 10^-3^
CDS	32,962	23,721	6.8 × 10^7^	3.86	2.7 × 10^-3^
3'UTR	20,264	1,143	5.0 × 10^6^	0.07	2.9 × 10^-4^

#### Size distribution of introns within UTRs and CDSs

**Figure 1:**
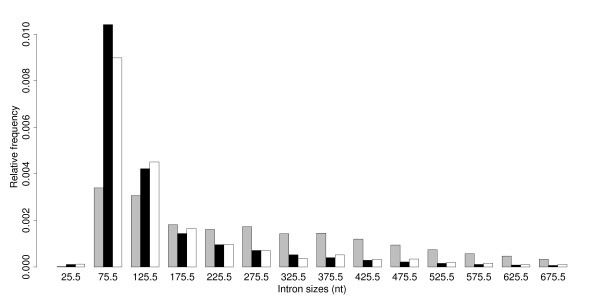
**Comparison of the length distributions of 5'UTR,3'UTR and CDS introns**. Bar graph comparing the length distribution of 5'UTR (grey), CDS (black) and 3'UTR (white) introns. The *x*-axis labels give the mid-points of the length range that each bar covers (e.g. if the range is 51–100 nucleotides, the mid-point is 75.5, bin size = 50 nucleotides).

Figure 1 shows the size distribution of introns within 5'UTRs, CDSs and 3'UTRs. The 3'UTR and CDS introns have very similar length average distributions (3'UTR: n = 1,468, mean = 208 nucleotides, median = 104 nucleotides, LQ = 88 nucleotides, UQ = 192 nucleotides, SD = 325 nucleotides; CDS: n = 127,141, mean = 158 nucleotides, median = 98 nucleotides, LQ = 85 nucleotides, UQ = 157 nucleotides, SD = 167 nucleotides). 5'UTR introns (n = 4,240, mean = 316 nucleotides, median = 253 nucleotides, LQ = 122 nucleotides, UQ = 416 nucleotides, SD = 292 nucleotides) are, on the other hand, significantly biased towards longer lengths (Kolmogorov-Smirnov test, 5'UTRs v. CDSs, D = 0.41, *p*-value < 10^-15^). Notably, there is a significant under-representation of short introns between 50 nucleotides and 150 nucleotides and a notable increase of introns above 200 nucleotides. These results are not expected to be greatly biased by incomplete UTR annotation. The significant differences between the size of introns that reside within the 5'UTRs compared to introns that reside within CDSs and 3'UTRs may be a feature that influences their role in enhancing translation levels. It is also plausible that the large 5'UTR introns function as spacers within the genome, providing an AT-rich stretch of sequence between coding sequence and promoter.

#### Intron position in UTRs

**Figure 2:**
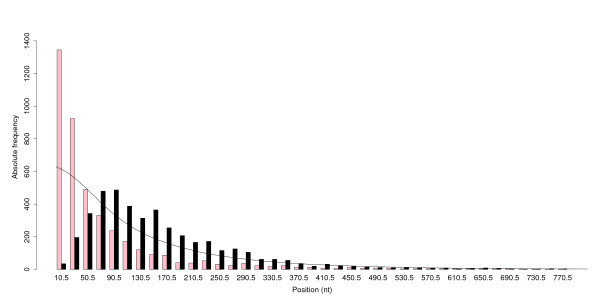
**Distribution of 5'UTR intron positions relative to the start and end points of the associated UTRs**. Pink bars represent the observed positions of 5'UTR introns relative to the end of the 5'UTR (i.e. the start codon ATG). Black bars represent the observed positions of 5'UTR introns relative to the beginning of the 5'UTR. The line represents the random model expected values (identical for both the black and pink bars). The positions are the intron excision points in the spliced transcript. The *x*-axis shows the mid-points of the length range that each bar covers (e.g. if the range is 41–60 nucleotides, the mid-point is 50.5, bin size = 20 nucleotides).

It has been suggested that the splicing of 5'UTR introns would lead to deposition of EJCs, which interact with translation initiation, resulting in translational enhancement [22-26]. If the EJCs facilitate the recruitment of ribosomes, then there may be selection on the position of introns within 5'UTRs; specifically there may be an optimal length between the position of the intron within the 5'UTRs and the translation start codon. Introns closer to the ATG would recruit the EJC and facilitate an interaction between the RNA, *trans*-factors and the ribosome. We compared the observed 5'UTR intron position distributions with the distributions that would be expected if introns were distributed uniformly (i.e. constant insertion probability after any given nucleotide) throughout 5'UTRs – calculated using Monte-Carlo simulations (see Methods). Figure 2 presents the distribution of intron positions within the 5'UTRs, relative to the beginning and end of the corresponding 5'UTRs. Clearly, the actual position distribution of 5'UTR introns relative to either the beginning, or the end, of the 5'UTR deviates from the expected distribution. It appears that the introns are more frequently located distant from the beginning of the 5'UTR (80–300 nucleotides after the start of the 5'UTR), and more frequently located proximal to the end of the 5'UTR (1–40 nucleotides before the start codon). Although incomplete 5'UTR annotation may result in some bias in the absolute positional distributions, since the Monte Carlo model distribution is based on the same 5'UTR sequences, the deviations from the expected distribution are real. The proximity of 5'UTR introns to the translation start site is consistent with a role that involves a function in translation, given the simple model of translation we are using. This could be achieved through alteration in the secondary structure of the 5'UTR leader either directly through the act of recruiting the spliceosomal complex during splicing or through the deposition of EJC proteins following processing. Recently the secondary structure of 5'UTRs has been shown to influence post-transcriptional regulation [40]; it is interesting to speculate whether the presence of an EJC could influence RNA folding and the consequences of this for gene regulation. Further experiments to alter the 5'UTR intron position will clarify if this has any biological role in post-transcriptional regulation.

#### Nucleotide conservation around the splice junctions

**Figure 3:**
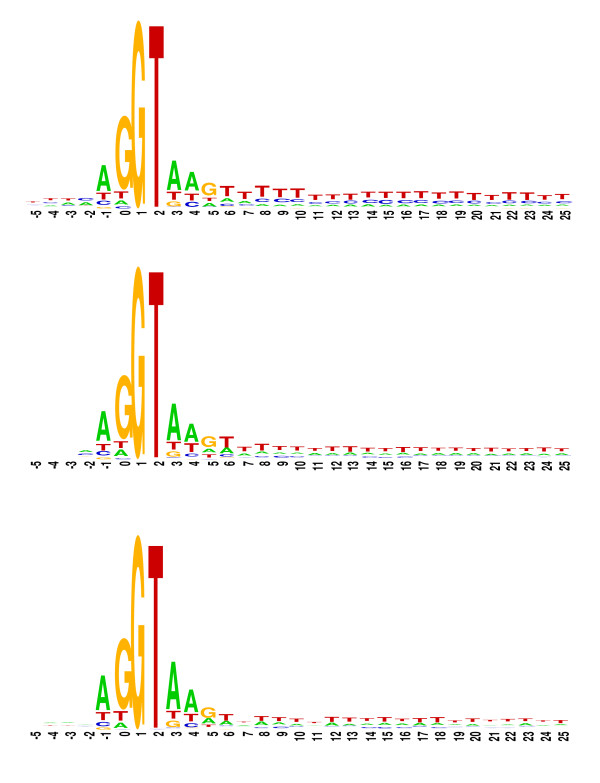
**Sequence logos showing the nucleotide bias around the donor site of 5'UTR, CDS and 3'UTR introns**. The *x*-axis refers to bases from the beginning of the intron, letter heights reflect the nucleotide bias at each position. Only 5 nucleotides of exon and 25 nucleotides of intron sequence at the donor site were included in the sequence logo as deviation of the nucleotide usage from background levels is not apparent outside these regions.

**Figure 4:**
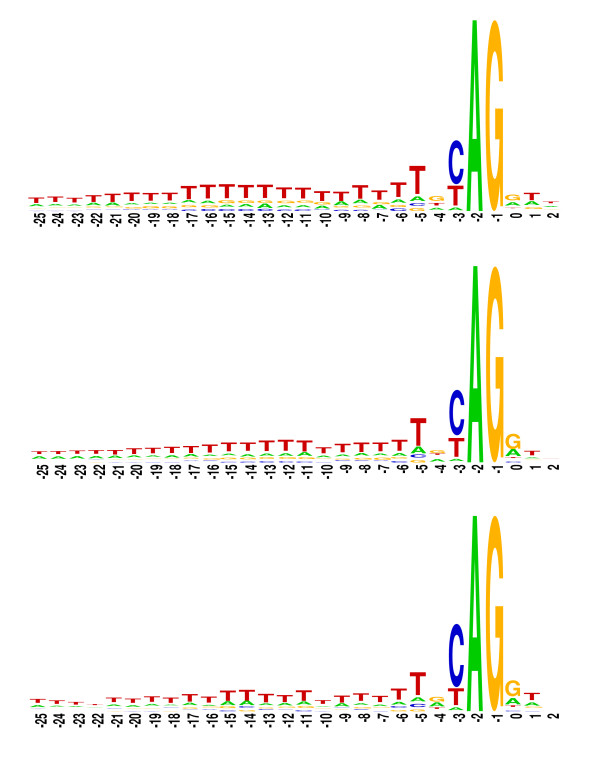
**Sequence logos showing the nucleotide bias around the acceptor site of 5'UTR, CDS and 3'UTR introns**. The *x*-axis refers to bases from the beginning of the intron, letter heights reflect the nucleotide bias at each position. Only 2 nucleotides of exon and 25 nucleotides of intron sequence at the acceptor site were included in the sequence logo as deviation of the nucleotide usage from background levels is not apparent outside these regions.

An investigation of the nucleotide preference around the splice junctions allows a more detailed comparison between the UTR introns and the CDS introns. Sequence logos [41] were created to visualize the nucleotide conservation around the splicing donor (GT) and the splicing acceptor (AG) junctions for each of the intron categories 5'UTR, CDS and 3'UTR (see Methods). The resulting logos are presented in Figure 3 (donor) and Figure 4 (acceptor). Only six and eight nucleotides of exon sequence from the donor and acceptor sites, and 25 nucleotides of intron sequence were included in each sequence logo, as there was no noticeable nucleotide bias outside these regions. The sequence logos shown in Figures 3 and 4 present all the canonical fingerprints of introns, namely the G/GT and AG/G splice site consensus, and the AT-rich element, which is a key feature for efficient splicing in plant introns [42,43]. There is, however, a significant C-rich region near the donor site of 5'UTR introns (+8 to +25 bases after intron start) (Figure 3) that does not appear to be present in either CDS or 3'UTR introns. There is also an increase in the occurrence of G and C residues in the region near the acceptor sites of 5'UTR introns (-17 to -8 bases before intron end) (Figure 4). These sequence biases could be necessary for the spliceosomal recognition of introns within non-coding sequence; however, these sequence biases are particular to the 5'UTR and are not seen within the 3'UTR, so their role cannot be general to non-coding introns. Further experimental analysis of the post-transcriptional and pre-translational effect of altering these sequences is necessary before any biological significance can be attributed to these observations.

### Molecular analysis of effects of introns in 5'UTRs on gene expression

**Figure 5:**
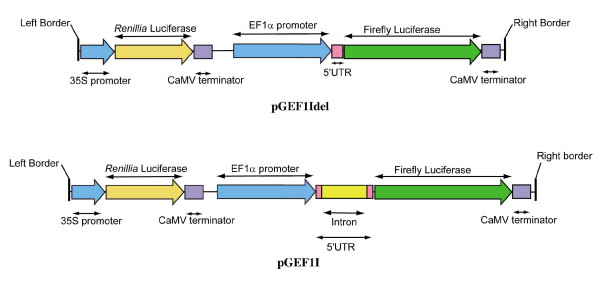
**Schematic diagram of the plasmids, namely pGEF1I and pGEF1Idel**. The plasmidswere used in the transient assays shown in Figure 6.

**Figure 6:**
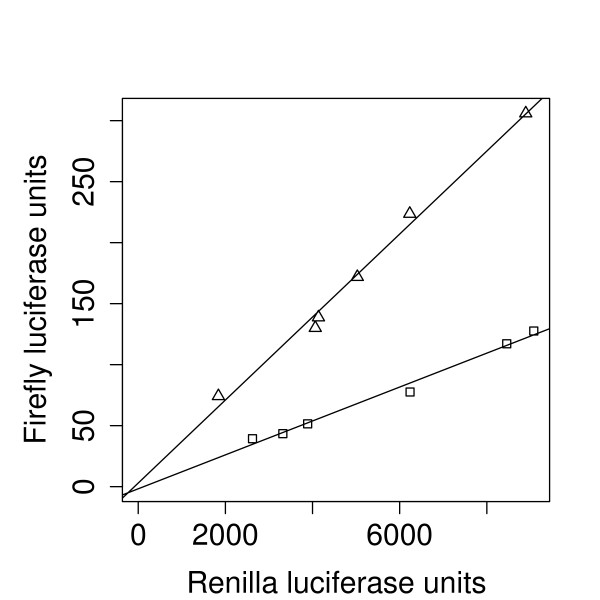
**Scatter plot of the actual firefly and *Renilla *counts**. Transient effect of 5'UTR intron (602 nucleotides) presence on the gene expression. The relative expression (± standard errors) is 0.0340 ± 0.0016 with the intron (triangles, pGEF1I) and 0.0139 ± 0.0008 without the intron (squares, pGEF1Idel).

In order to investigate the effects of 5'UTR introns on gene expression, the annotated intron-containing 5'UTR of EF1α-A3 (AT1G07940) from *A. thaliana *was fused to the firefly luciferase reporter gene in the Ti binary vector pGreenII 0800-LUC [44], resulting in plasmid pGEF1I (Figure 5). In addition, each 5'UTR was modified to represent an intronless version of the original pGEF1Idel (Figure 5). In removing the intron, the exon sequence of the 5'UTR was not altered and resembled an accurately processed 5'UTR. The resulting plasmids were then analyzed via *Agrobacterium*-mediated transient infiltration into *Nicotiana benthamiana *leaves, followed by dual-luciferase assay [44]. The assay system measures the enzyme activity of the experimental reporter, firefly luciferase (LUC), as well as the enzyme activity of the control reporter, *Renilla *luciferase (REN), which provides an internal control and is under the transcriptional regulation of the 35S promoter. Thus, the activity of the LUC can be normalized by the activity of the REN, in order to minimize experimental variability such as differences in expression caused by different plants, leaf age and infiltration volume. Figure 6 presents the relative luciferase assay of pGEF1I and pGEF1Idel, and is a typical dataset from a dual-luciferase assay: here six independent infiltrations were assayed for each of the plasmids under investigation. As the data fit a linear regression well (R^2 ^= 0.95), the regression gradient and its standard error were taken as the measurement of reporter gene activity. The relative expression of pGEF1I is 0.0340 ± 0.0016 and pGEF1Idel is 0.0139 ± 0.0008, indicating a significant (*t*-test, *p*-value = 6.3 × 10^-8^) enhancement of reporter gene activity under the presence of the 602-nucleotide 5'UTR intron. This data is consistent with IME requiring transcription, and acting post-transcriptionally on RNA to influence either stability or translatability.

#### Confirmation of transient assay data in stable transgenic *A. thaliana*

**Figure 7:**
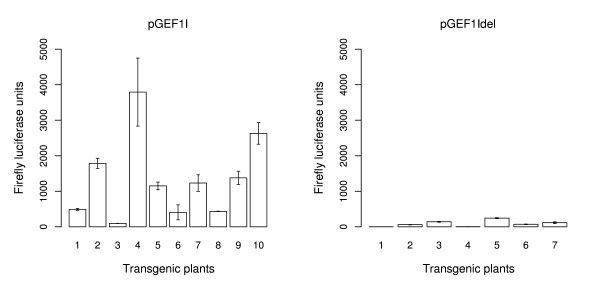
**Absolute firefly luciferase activity of transgenic plants**. Average of absolute firefly luciferase activity for two T1 leaves from each of 10 independent plants transformed with pGEF1Ikan, and 7 independent plants transformed with pGEF1Idelkan. Error bars are standard deviation of two T1 leaves from the same plant.

We also generated transgenic *Arabidopsis *to confirm the level of IME obtained in transient assay data for the pGEF1I and pGEF1Idel fusions. The plasmids used for our transient assay were modified to contain the plant kanamycin transformation selection gene and transformed into *A. thaliana *'Columbia' by the floral dip method [45]. Figure 7 shows the absolute LUC activity for two leaves from each of 10 independent T1 plants transformed with the intron-containing EF1α-A3 5'UTR, and from each of 7 independent T1 plants transformed with the intronless EF1α-A3 5'UTR. In all these transgenic lines, the *Renilla *luciferase gene was inactive, despite being under the transcriptional control of the 35S promoter. Therefore the relative expression of LUC was not determined. It is not unusual for some reporter genes to become inactivated during the transformation process [46], though zero out of 17 is unusual and may reflect a higher susceptibility of the *Renilla *luciferase gene to this form of gene-silencing. This transgene silencing is probably because of a post-transformation event, such as methylation-mediated transcriptional silencing [47]. Nonetheless, although there is much variability in the absolute levels of LUC activity, expressed as relative light units per mg of leaf fresh weight, there is a striking elevation in the level of LUC expression from those transgenic lines transformed with an intron in the 5'UTR relative to those without a 5'UTR intron (*t*-test, *p*-value = 0.0037). This is consistent with the transient expression data. These T1 lines were not analyzed for T-DNA copy number, so some of the variability between transgenic lines containing the same construct could be due to the number of T-DNA insertions in each of the transgenic lines.

#### Effect of a viral suppressor of silencing on intron-mediated enhancement

Post-transcriptional gene silencing (PTGS) or RNAi is a mechanism that is used to degrade RNA transcripts after they have been transcribed [48]. PTGS is activated by dsRNA to produce siRNA that can act as signalling molecules, promoting a cascade of mRNA degradation [49,50]. As IME is a post-transcriptional regulation, it is possible that the inclusion of certain introns within the 5' region of a gene reduces the RNA's susceptibility to siRNA, through some unknown mechanism. In order to assess the involvement of PTGS in IME, a similar transient assay using plasmids pGEF1I and pGEF1Idel (Figure 5) was performed using a modified version of the pSoup helper plasmid that is able to suppress gene silencing [44]. To suppress gene silencing the p19 expression cassette from pBIN 61-P19 was cloned into pSoup and therefore resident within the same *Agrobacterium *cell as the dual-luciferase reporter cassette. The P19 enzyme is a viral silencing suppressor from the tomato bushy top virus (TBTV), which prevents activation of PTGS [51]. Although gene expression of both the LUC and REN reporter genes are enhanced when co-expressed with P19, both with and without the intron, under the presence of P19 (0.2185 ± 0.0183 with intron and 0.0692 ± 0.0073 without intron) the enhancement achieved by the presence of the 5'UTR intron was consistent with the relative enhancement that is obtained without P19 (0.1089 ± 0.0081 with intron and 0.0315 ± 0.0034 without intron). As the IME levels in the presence and absence of P19 are similar, we conclude that no component of this IME can be attributed to PTGS.

#### Partial deletion of the EF1α-A3 5'UTR intron

**Figure 8:**
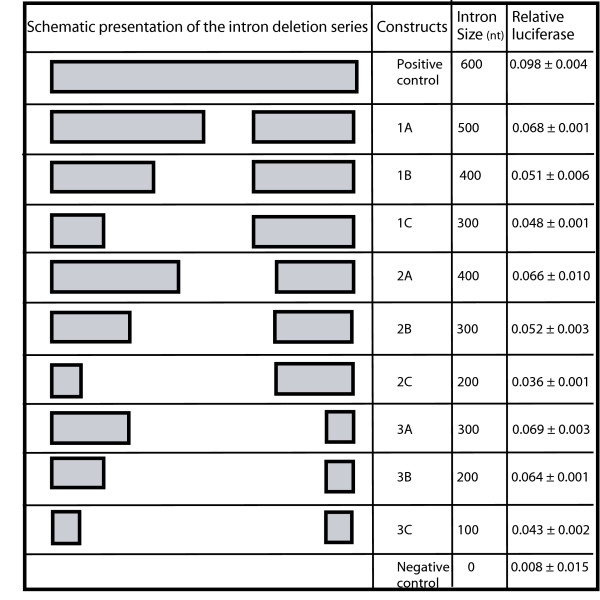
**Schematic diagram of AtEF1α-A3 5'UTR intron deletion series**. A series of truncated EF1α-A3 5'UTR introns from AT1G07940 were generated. Fifty nucleotides up and downstream of the intron acceptor and donor sites were maintained for splicing efficiency. The deletions are grouped according to the position of the deletion point relative to the 3' region of the intron. Normalised relative luciferase values represent the regression of six independent measurements (see also Figure 9).

**Figure 9:**
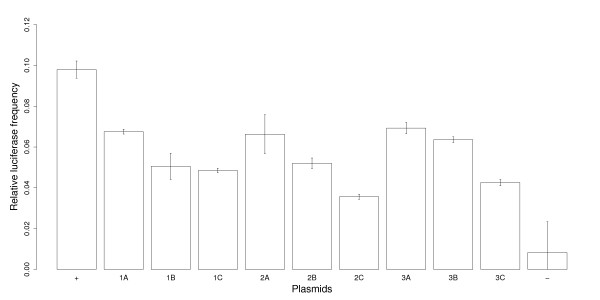
**Luciferase expression of AtEF1α-A3 5'UTR intron deletions**. On the *x*-axis, the number below each bar indicates the plasmid tested (Figure 8). Each bar represents the regression from six independent LUC-REN expression measurements. Error bars are standard errors.

One of the most prominent observations from the bioinformatics analysis was the dramatic difference in the length distribution of 5'UTR introns compared with both CDS and 3'UTR introns (Figure 1). To test the influence of 5'UTR intron size on post-transcriptional enhancement, a series of truncated EF1α-A3 5'UTR introns were generated. Fifty nucleotides up and downstream of the intron acceptor and donor site were maintained for splicing efficiency so as not to interfere with the possible role of intron sequence proximal to these splicing junction sites. Figure 8 is a schematic representation of the nine intron deletions used in this experiment, grouped according to the position of the deletion point relative to the 3' region of the intron. Figure 9 presents the relative luciferase activity for each of these constructs, along with the complete intron, and the intronless reporter-LUC fusions. Comparing the differences in reporter gene expression for each series of constructs that retain the same 3' portion of the intron sequence (i.e. constructs 1A-C, 2A-C, and 3A-C), shows a clear positive correlation between gene expression and intron length: the longer the intron, the greater the relative level of LUC activity (*t*-tests: 1ABC *p*-value = 2.8 × 10^-5^; 2ABC *p*-value = 6.7 × 10^-5^; 3ABC *p*-value = 4.0 × 10^-4^). On the other hand, if constructs are grouped by their 5' end, there is no obvious correlation between intron length and LUC activity.

These observations suggest that IME in EF1α-A3 is mediated by, not one, but three or more elements distributed over the 5' 350 nucleotides of the 5' UTR intron, with relatively little effect from the 3' 250 nucleotides. Indeed, multiple AT-rich stimulatory elements have been previously described in plants [10,30-33]. and, consistent with this, the EF1α-A3 5' UTR intron is AT-rich. It is interesting that the three inferred IME elements – distributed over 300 nucleotides – require a very significant 'increase' in intron length when compared with the median lengths (CDS introns 98 nucleotides; 5'UTR introns 253 nucleotides). Potentially, this offers an explaination as to why the median 5'UTR intron length is so much greater than the median CDS intron length, although experiments on more genes would be required to confirm this.

## Conclusion

A growing number of plant expression studies have revealed that the presence of an intron within the 5'UTR induces enhanced RNA and protein accumulation. However, the intrinsic determinants of 5'UTR IME in plants, especially the role of any sequence motifs within the intron, remain poorly defined. In this paper, we have presented extensive statistical analyses of all the annotated *A. thaliana *5'UTR introns in the TAIR database and shown that 5'UTR introns are noteworthy in terms of their nucleotide composition around the splicing donor and acceptor site, the distribution of intron sizes and the position distribution within the UTR and proximity to the ATG start codon. In addition, we have shown that, not only can the presence of an intron in the 5'UTR significantly enhance gene expression in at least one gene, but the length of intron also influences the level of gene expression. These results should be beneficial in determining the mechanism of IME in plants, as well as determining the origin and role of 5'UTR introns. As these introns are not embedded within coding sequence, the flanking nucleotides can be modified without interfering with the open reading frame (cf. CDS introns). We believe that this makes the introns that reside within non-coding sequences a powerful resource to assist in unravelling the role of introns within the genome.

## Methods

### TAIR database

In this study, the statistics of *A. thaliana *introns were analysed using the TAIR (The *Arabidopsis *Information Resource) database [18,55]. The 25/03/2005 version was used (with corrected reverse-strand nucleotide coordinates). The TAIR database was utilized instead of the GenBank database [52] for several reasons, including (1) the DNA sequences in the TAIR database employ data from all of the GenBank, AtDB (Arabidopsis thaliana Data Base), and TIGR (The Institute for Genomic Research) [37], and (2) the quality of GenBank entries is not uniform and many sequences are not supported by cDNA and EST data, whereas the TAIR entries enjoy support from a variety of sources. The files extracted from the sequence database on the TAIR FTP site are listed in Table 2.

**Table 2:** Files extracted from TAIR FTP site [55]. Due to the small amount of miss-annotation that may interface with the bioinformatics analysis, UTRs less that 4 nt in length and introns less than 6 nt in length were excluded from the analysis.

File	Type of data
ATH1_3_UTR_20050325	Coordinates and sequences of 3'UTRs
ATH1_5_UTR_20050325	Coordinates and sequences of 5'UTRs
At_intron_20050330	Coordinates and sequences of introns
Sv_gene_feature.data	Coordinates of CDSs, ORFs, exons and genes
ATH1_chr1.1con.01222004	Chromosome 1 – complete sequence
ATH1_chr2.1con.01222004	Chromosome 2 – complete sequence
ATH1_chr3.1con.01222004	Chromosome 3 – complete sequence
ATH1_chr4.1con.01222004	Chromosome 4 – complete sequence
ATH1_chr5.1con.04172003	Chromosome 5 – complete sequence

### Data analysis

Data were processed using C-shell scripts, C++ programs, and the statistics package R version 2.0.1 [53]. Basic statistics (means, standard deviations, regression, etc.) and statistical tests (*t*-tests, Kolmogorov-Smirnov tests etc.) were calculated using R. Also, Figures 1, 2, 6, 7 and 9 were drawn using R.

The Monte-Carlo simulation program – written in C++ – was used to calculate the expected position distribution of 5'UTR introns if introns were distributed uniformly (i.e. constant insertion probability after any given nucleotide) throughout 5'UTRs. To do this, all of the original introns were extracted from all of the original 5'UTRs, and then they were randomly re-inserted into the processed 5'UTR sequences. This was repeated 10,000 times, and the average intron position distributions were calculated. Sequence logos were drawn using WebLogo version 2.8 [41]. The input sequences were extracted from the chromosome files [Table 2] using a C++ program, and the nucleotide frequency matrices around splice junctions were calculated using C-shell scripts.

### Isolation of Arabidopsis 5'UTRs

The 1.87 kb promoter of the *Arabidopsis *EF1α-A3 (AT1G07940) gene was isolated from genomic DNA of 'Columbia' using primers RPH-130 (TCTAGAATGGTACCTAATTACTTCAC) and RPH-131 (CTCTTTACCCATGGTTAGAGACTG). The PRH-130 primer altered the sequence at the 5' end of this promoter, introducing a *Kpn*I site as well as an *Nco*I site at the ATG of this gene. The PCR product was cloned into pGEM-T Easy (Promega) and sequenced to ensure accurate amplification. Similarly, four other promoters were isolated: AT1G10670 (0.651 kb) CAS-001 (GGTACCCACAAATGGAATGGTTGAAG) and CAS-002 (CTTCCTCGCCATGGCAAAACGAAAACTGG); AT1G13980 (2.1 kb) CAS-013 (GGTACCTAGAGGTGTGTATGATAATG) and CAS-014 (CCATGGAATCTGCTCAAATCTTCAGCCAG); AT1G17470 (0.92 kb) CAS-017 (GGTACCTGTAGCGTTTCTACTCTCGT) and CAS-018 (CCATGGTGCTTCACTTGTTTTTGC); AT1G72050 (2.7 kb), CAS-021 (GGTACCATTCGGTCACTGAAGACAC) and CAS-022 (CCATGGTGCGTGATCGAGGCTTACTTGC). In all cases, a *Kpn*I site was introduced at the 5' end of the promoter and an *Nco*I site at the ATG.

### Modification of promoter clones

Intron-containing promoter-UTR clones were modified to become intronless by a version of inverted PCR. In the case of AtEF1α-A3 (AT1G07940), two primers were utilized. A forward primer, RPH-133 (CTCAGAGATATCGCAAGAGAG), corresponded to the region of the sequence at the 3' end of exon I, the sequence that precedes the 5'UTR intron; this primer is homologous to the complementary strand and primes towards the upstream promoter region. A reverse primer, RPH-134 (ATTTGTTTGACAGTCTCTAAC), corresponded to the 5' region of exon II, the sequence that directly follows the 5'UTR intron. These two primers were used in a PCR amplification with *Pwo *polymerase (Roche), using the pGEM-T Easy clone of the intron-containing promoter as template. PCR products were then treated with polynucleotide kinase (NEB) and re-circularized with T4 DNA ligase (NEB). Because of the blunt termini created by the *Pwo *polymerase, the ligated product excludes the intervening sequence between the divergent primers and, in our case, precisely removed the intron sequence as it would be in the processed mRNA. In the same way, intronless versions for 4 other *Arabidopsis *promoter clones were generated: AT1G10670, CAS-003 (CGTTGAGAGAGAATGGGGGTAG) and CAS-004 (TTTTCGTTTTGCCATGGCGAGG); AT1G13980, CAS-015 (TGTTTCTCCAGCGATTCAGAG) and CAS-016 (TAATGATTGAGTTTGGCCTCTATC); AT1G17470, CAS-019 (AGTGGAATTGGTGAAGGGCG) and CAS-020 (ATAGTAGCAAAAACAAGTGAAGC); AT1G72050, CAS-023 (CTGATGATGAGATTGGATGTG) and CAS-024 (ATGGAACTTGAAGAGGAAAGAG) for intron 1, CAS-025 (CTCGAGCGAATGACTCTGCA) and CAS-026 (GAAATAAATAGCCTTTTGTTT) for intron 2. As AT1G72050 has two 5'UTR introns, two rounds of intron-out-PCR were needed to generate the intronless version of this promoter.

### Construction of dual-luciferase reporter cassette

Promoter fragments were subcloned into the binary vector pGreenII 0800-LUC [44]. This vector includes the *Renilla *luciferase (Promega) reporter gene (REN) under the transcriptional regulation of 35S promoter and CaMV terminator [54], and a promoter-less firefly luciferase LUC (Promega) with a CaMV terminator. The 5' end of the firefly luciferase contains multiple cloning sites suitable for the insertion of promoter fragments forming translational fusions. Binary vectors were electroporated into *Agrobacterium *GV3101 (MP90) according to [54]. As the initiating ATG of firefly luciferase has an *Nco*I site (CCATGG), the fusion between promoter-5'UTR and reporter gene contains no intervening sequence. The intron-containing and intronless constructs containing the AtEF1α-A3 promoter were converted to stable plant transformation vectors by inserting a nos-kan selection cassette [54] downstream of the LUC reporter gene.

### Generation of EF1α-A3 intron deletion series

Deletions within the EF1α-A3 intron were achieved by performing the same PCR method used to remove the whole 5'UTR intron described above. A combination of diverging primers to the 5' (primer A, B or C) and 3' (primer 1, 2 or 3) of the deletion point were used in generating nine intron deletions. Primer A (RPH-258, GATCAACAGAAGAGAAAGAAGCA), Primer B (RPH-259, CACCACAGATCAGAAATTCCAAA), Primer C (RPH-260, GAACCAGATCGATCATATAGTTTA), Primer 1 (RPH-261, AAGTCTACTGTTTTTCTTGATTC), Primer 2 (RPH-262, AGGGTCGCTTAGCTCAGTTGATA), Primer 3 (RPH-263, AGCATAAACAATCAATTGATTCA).

### Transient analysis of firefly and Renilla luciferase

Reporter gene plasmids were electroporated into *Agrobacterium *GV3101 (MP90) [54]. *Agrobacterium *were cultured in Lennox agar (Invitrogen) with 50 mg/ml kanamycin (Sigma) at 30°C for 3 days. Cells were re-suspended in infiltration media (10 mM MgCl_2_, 10 μM acetosyringone) until OD^600 ^= 0.2 and allowed to incubate at room temperature for 2 hours prior to infiltration. Re-suspended *Agrobacterium *were infiltrated into the leaves of 3–4 weeks old *Nicotiana benthamiana *(16 h day length, 22°C) and the plants were allowed to grow for a further 3 days. Infiltrated patches were ground in 500 μL Passive Lysis Buffer (PLB) (Promega), diluted (5:500 in PLB), and 5 μ'L used for dual-luciferase assay using the Dual-Luciferase Reporter (DLR™) Assay System (Promega). Relative light units (RLU) were measured over 15 seconds following 5 seconds of delay with a Turner 20/20 luminometer. Relative luciferase activity was calculated by performing a regression analysis from 6 independent measurements using the statistics package R version 2.0.1 [53].

### Transgenic A. thaliana

The transgenic *A. thaliana *'Columbia' were created via the floral dip method [45] with the two stable plant transformation vectors containing the AtEF1α-A3 promoter and a nos-kan selection cassette.

## Authors' contributions

BYWC: bioinformatics, data analysis and manuscript preparation. RPH: experimental design, EF1α-A3 intron deletions, transient assays data analysis and manuscript preparation. CS: isolation of *Arabidopsis *promoters, intron deletions and generation of stable transgenic plants. AEF: bioinformatics. CMB: supervision of BYWC. All authors read and approved the final manuscript.
